# How to use telesimulation to reduce COVID-19 training challenges: A recipe with free online tools and a bit of imagination

**DOI:** 10.15694/mep.2020.000129.1

**Published:** 2020-06-22

**Authors:** Carla Sa-Couto, Abel Nicolau

**Affiliations:** 1 Biomedical Simulation Center (CSB-FMUP); Center for Health Technology and Services Research (CINTESIS); Faculty of Medicine of University of Porto (FMUP)

**Keywords:** Telesimulation, Emergency Scenarios, Non-technical Skills, COVID-19, Free tools, Online Resources, Recipe

## Abstract

This article was migrated. The article was marked as recommended.

In response to COVID-19 outbreak, alternatives to face-to-face classes/seminars rapidly emerged and telecommunication platforms become the new classrooms. Although with additional challenges, telesimulation based classes can benefit from online platforms and innovative strategies, to promote a meaningful and interactive distance-learning experience.

A combination of several free online resources is used to recreate (at distance) emergency simulation scenarios. Practicalities involving this strategy are described in the form of a recipe, including supplemental guides and an illustrative video, allowing its rapid implementation. Feedback from students on the educational gains was surveyed, as a preliminary evaluation of this methodology.

The use of free online tools makes it accessible off-site and worldwide, including in low-resources locations. This strategy has high potential to be used in the transitional post-confinement period or as a future complement to physical settings.

## Background

Telesimulation can be defined as a process in which telecommunication and simulation resources are used to provide education, training, and/or assessment to learners at an off‐site location ( McCoy, 2017). Telesimulation is being used as a distance-education strategy for over a decade (von Lubitz, 2003; Haile‐Mariam, 2005), with an increased interest in the recent years. It can be used to develop two of the three domains of learning ( McCoy, 2017): cognitive (knowledge) and affective (attitudes).

In a typical telesimulation class, learners are in a location physically distant from the academic facilities (eg. home) with no (or very limited) educational or training resources, while the teacher/instructor is in a simulation center or in a learning facility with (physical) educational resources.

COVID-19 outbreak has suddenly disrupted the world, demanding immediate adjustments to all dimensions of people’s life. Medical education is no exception. Throughout the globe, universities are closed and classes suspended, with recommendations of prophylactic confinement to all not involved in patient care or first-need services. Alternatives to face-to-face classes rapidly emerged and telecommunication platforms are the new classrooms.

Telesimulation encounters also need to readjust, considering learners and teachers are off-site, with very limited educational resources, at both ends. Although with additional challenges, telesimulation based classes can benefit from online platforms and innovative strategies, to promote a meaningful and interactive distance-learning experience.

This article describes a combination of several free online resources used to recreate (at distance) emergency simulation scenarios. Practicalities involving this strategy are described in the form of a recipe, allowing its rapid implementation.

## The recipe

For each scenario, you will need:

• an instructor/facilitator
• a simulation technician (or a second instructor)
• students q.b.

### Ingredients

#### 1. Teleconferencing platform.

Zoom Video Communications, Inc. ( https://zoom.us/) is a free teleconferencing platform. It is used worldwide and was selected because it includes a number of useful features (described in detail in ***Supplement File 1***).

#### 2. An online serious game for setting the scene.

Full-Code App ( https://app.full-code.com/Player/Player.html) is a serious game that simulates an emergency room. It offers 7 free clinical cases, with many other cases available for buying. The case itself is not relevant for your specific scenario, as it will be used just to create the environment (setting the scene).

#### 3. A software that simulates a vital signs monitor.

VitalSignSIM is a software developed by our institution that emulates a simple vital signs monitor. It allows easy control of the signals through the computer keyboard. It is free and available for download at: https://simulacao.med.up.pt/tools-resources/.

#### 4. Computers/tablets and cell phones.

The staff (instructor and technician) should have 2 computers/tablets each. One computer will be the main control station, while the other will allow the observation of students’ view (to monitor what is displayed). The staff should also have their cell phones available (with headphones and built-in microphones), to assure a private communication channel.

#### 5. Instructions and recommendations.

Documents with clear instructions and recommendations must be created for staff and students.

#### 6. Prompts and cues.

For each scenario, a set of illustrative images (imagology, physical signs, etc) and/or documents (patient charts, lab results, etc) should be created/available.

#### 7. A scenario script.

It is suggested to use a well-known scenario(s) to diminish the cognitive burden for both the instructor and the technician.

#### 8. Time and dedication.

The implementation steps require dedication and time to adapt to the inherent technologies and processes.

*Please note* *:* These were the “ingredients” tested and used. Other “ingredients” (i.e other telecommunication platform or simulation software) can also be used and combined, with concomitant adjustments in the preparation.

### Preparation

**Figure 1.**
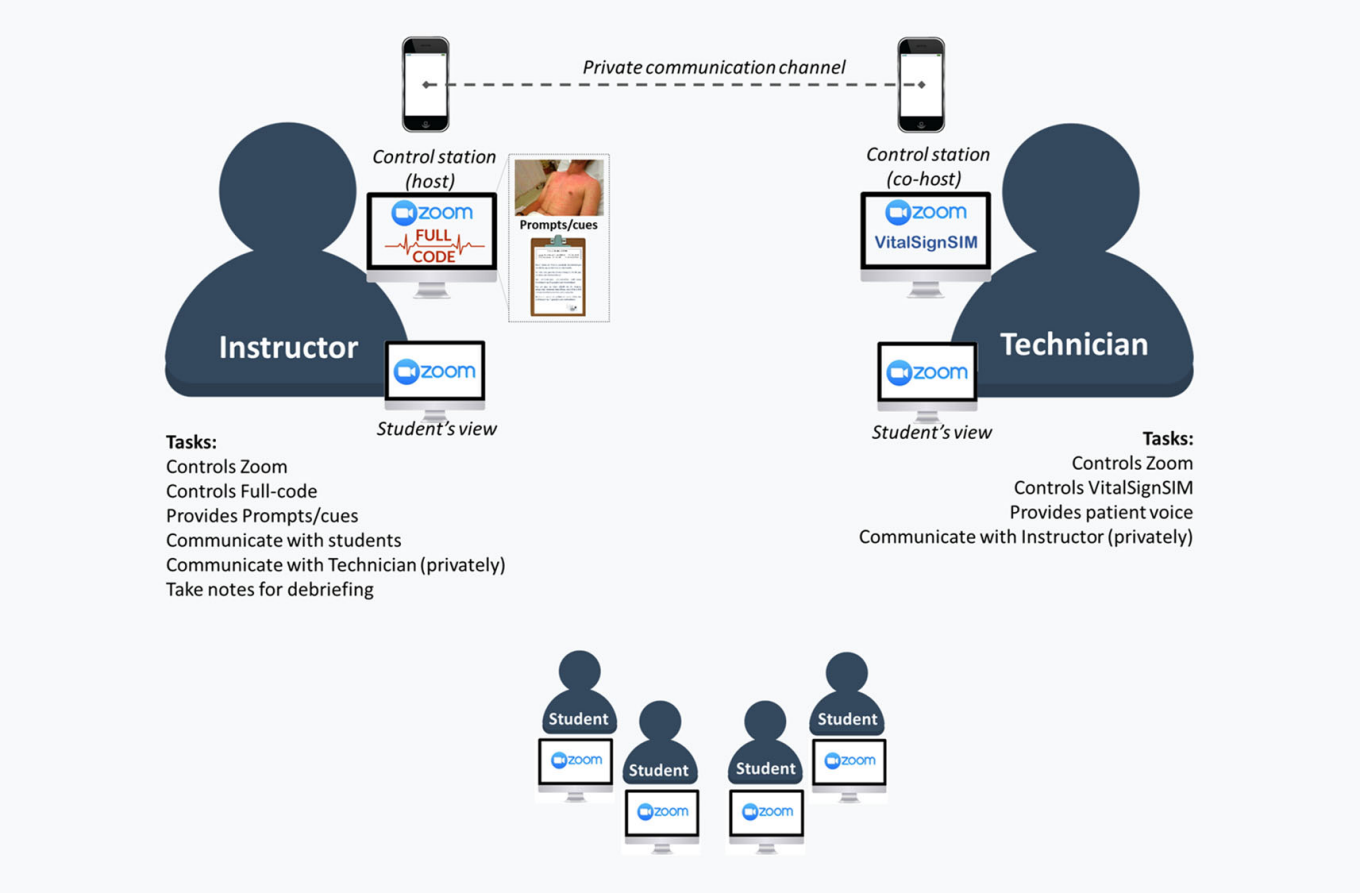
Elements involved in the proposed telesimulation setting. Required equipment and software allocation is represented through generic icons and software logotypes. Also included: overall tasks of instructor and technician.

• Install all necessary software, as follows ( Figure 1):
  • Zoom should be installed in all 4 computers/tablets; Students also need to install Zoom;
  • VitalSignSIM needs to be installed in control station of the technician;
  • Full-Code App does not need installation as it runs on a website. It should run from the instructor control station.

• If you are not familiarized with the software and the teleconferencing platform, dedicate some time (a couple of hours) to test it.

• Prepare a configuration guide with clear steps for the staff (instructors/technicians). Clearly indicate the tasks attributed to each element, in a chronological order. Include illustrative images. An example of a configuration guide (including detailed settings for Zoom and tips and tricks for the selected simulation software) is available in ***Supplement File 1***.

• Prepare an orientation guide with recommendations and instructions for students. This guide should address the technical aspects but also the ground rules for their participation in the scenario (e.g. be in a private, quite space; verbalize everything you need; etc) and recommendations to promote an immersive scenario (e.g. make gestures with your hands when performing a technical task; wear your white coat; etc). An example of an orientation guide is available in ***Supplement File 2***.

• Review the scenario and identify prompts and cues needed to be prepared. These are essential to provide information to students and will replace physical cues. For example, prepare an image with a cutaneous rash for an anaphylactic shock scenario.

• Make a thoroughly rehearsal with your staff. Start with the configuration settings (use the configuration guide). For the scenario itself, rehearse how you will welcome your participants (e.g. select a specific view - room entrance) and the assignment of students to breakout rooms (if applicable). Run the scenario, including typical (un)expected actions, patient voice, prompts and cues. Use the student’s view computer to monitor what students will observe.

• Make re-adjustments, if needed, and rehearse again.

• Brief your participants. As in the simulation center, a briefing of the “space” and “simulator” is essential. Use the orientation guide to review all rules and expectations.

• Prepare a feedback survey. This is a new approach that will benefit from updates and improvement, based on participant’s experience.

## Serving the meal: implementation of telesimulation scenarios

The implementation of the proposed strategy occurred in April 2020, using a scheduled elective curricular unit as a pilot. This is a course for 5 ^th^ year medical students focused on the use of non-technical skills to promote effective teamwork, during clinical emergencies. Typically, it includes an introductory lecture (week 1), several group dynamics (week 2), and students’ participation (in groups of 4) in three emergency scenarios, followed by a structured debriefing (week 3 and 4). Although all course components were delivered/facilitated through teleconferencing, the major challenge (and the focus of this article) was the adaptation of the simulation scenarios through telesimulation.

The premise was to maintain the course as close as possible to the original. The three scenarios (and learning objectives) used in previous editions (gastro-intestinal bleeding, anaphylactic shock, and opioid overdose), were maintained. The typical structure (scenario + debriefing) was used with standard timings (15 min + 45 min, respectively). Eight students participated in this course. All students (in groups of 4) actively participated in all scenarios. At the end of the course, students answered a brief survey on the experience.

To illustrate how the instructor/technician conduct the scenario and how students intervene, **an annotated video** (student’s view) with parts from an actual scenario is available at: https://bit.ly/2TeeyP8.

A written informed consent declaration was signed by participants authorising the use of data and recordings.

## Did you enjoy the meal? - Preliminary assessment

Student’s feedback showed an overall positive reaction, with all (100%) agreeing that this was a valuable educational experience. Although they preferred physical scenarios, all (100%) agreed that telesimulation provides a comparable experience to physical scenarios. All (100%) agreed that the combined technologies were adequate and provided realism throughout the scenario. All but one (88%) considered essential the pre-scenario information and orientation guide, enabling an augmented performance.

All (100%) students agreed that this class allowed the acquisition/maintenance of non-technical skills and stated that they will change/improve their behaviour/action because of this course. Communication strategies (ISBAR, close-loop, think-aloud and summarizations) (100%) and leadership (distribution of tasks and team organization) (63%) were the skills pointed as easily trained. As expected, the physical actions (physical examination and the execution of tasks) were pointed as the most difficult to be trained (75%).

All students (100%) would like to have more classes using telesimulation, and all (100%) agreed that this strategy could be useful as a complement to face-to-face classes, post-COVID pandemic. Two students (25%) pointed as beneficial the limitations inherent to telesimulation, enabling the focus and amplification of communication skills.

## Final remarks

COVID-19 pandemic forced new and rapid adjustments to medical education and clinical training. Telesimulation can be used to promote a meaningful, interactive simulation environment, even with limitated physical resources. This article suggests a combination of free available online tools, making it accessible off-site and worldwide, including low-resources locations. Other tools (free or commercial) and combinations can be explored and used, with expected similar results.

This strategy has high potential to be used in the transitional post-confinement period or as a future complement to physical settings.

## Take Home Messages

• COVID-19 outbreak has suddenly disrupted the world, demanding immediate adjustments.
• Telesimulation can promote a meaningful and interactive distance-learning experience, even with limitated physical resources.
• We share a recipe for a rapidly implementation of this strategy, including supplemental guides and an illustrative video.
• The use of free online tools makes it accessible off-site and worldwide, including in low-resources locations.
• This strategy has high potential to be used in the transitional post-confinement period or as a future complement to physical settings.

## Notes On Contributors

**CARLA SÁ-COUTO**, PhD, is an affiliated professor, simulation educator and director of the Biomedical Simulation Centre of the Faculty of Medicine of University of Porto (BSC-FMUP), and senior researcher at the Centre for Health Technology and Services Research (CINTESIS). She is a member of the European Simulation Instructors Group (EUSIM), being the Portuguese delegate. She is currently serving as scientific committee chair of SESAM - Society for Simulation in Europe. Her main research interest is the use of simulation in medical education to improve non-technical skills and patient outcomes. ORCID ID: https://orcid.org/0000-0002-7616-0786

**ABEL NICOLAU**, MSc, is a biomedical engineer and simulation technical coordinator of the Biomedical Simulation Centre of the Faculty of Medicine of University of Porto (BSC-FMUP), and researcher at the Centre for Health Technology and Services Research (CINTESIS). His main research interest is the development of new technologies for simulation-based training and its impact in learning outcomes. ORCID ID: https://orcid.org/0000-0001-7683-5467

## Appendices

See Supplementary Files:

Suppl File 1 - Telesimulation Configuration Guide (staff ).pdf

Suppl File 2 - Telesimulation Orientation Guide (students).pdf
