# Supplementary material for: How to use telesimulation to reduce COVID-19 training challenges: A recipe with free online tools and a bit of imagination
Source: MedEdPublish (2016). 2020 Jun 22;9:129. [Version 1] doi: 10.15694/mep.2020.000129.1 (PMC10702647; doi:10.15694/mep.2020.000129.1)
Supplement: Peer review history

# Reviewer report: Reviewer response for version 1

**DOI:** 10.21956/mep.20094.r30958

**Published:** 2020-06-25

**Authors:** Felix Silwimba

**Affiliations:** 1 University of Lusaka

This review has been migrated. The reviewer awarded 5 stars out of 5 this article demonstrates innovation at its best . it demonstrates use of various approaches to deliver medical education.

Reviewer Expertise:

NA

No decision status is available

# Reviewer report: Reviewer response for version 1

**DOI:** 10.21956/mep.20094.r30959

**Published:** 2020-06-24

**Authors:** P Ravi Shankar

**Affiliations:** 1 American International Medical University

This review has been migrated. The reviewer awarded 4 stars out of 5 This is an interesting article about the use of telesimulation in a medical school in Portugal. The authors have provided a comprehensive description of the telesimulation module. The two supplementary files introduce the reader and helps him/her prepare for the scenario. The video was extremely useful and provides a visual representation of the scenario and the different steps involved. Some aspects like developing communication and leadership skills were not addressed in the video most probably due to student privacy issues. The authors also mention that they have developed the scenario using freely available tools and hence their recipe may be applicable to low resource settings also. I liked the use of the cook book scenario in this article. Telesimulation will have an important role to play in the post-COVID world as mentioned by the authors. Interprofessional collaboration is also evident in the module.

Reviewer Expertise:

NA

No decision status is available

# Reviewer report: Reviewer response for version 1

**DOI:** 10.21956/mep.20094.r30957

**Published:** 2020-06-23

**Authors:** Subha Ramani

**Affiliations:** 1 Harvard Medical School, Brigham and Women's Hospital

This review has been migrated. The reviewer awarded 3 stars out of 5 The authors have done an excellent job in their comprehensive and step by step description of how other educators might construct cases for tele simulation based education. For those educators who wish to design a similar curriculum, these instructions are very clear and cover all the steps needed from technology needed, planning, details of case development, brief evaluation of impact etc. The combination of an educator and an engineer is likely very valuable as their expertise will complement each other. The AV materials are of very good quality.While the recipe part is very well written and described, the introduction is very brief and no learning theories have been applied. A I more systematic and deliberate introduction with a problem statement, could enhance the scholarly approach.Similarly, a slightly more detailed discussion including next steps and a more extensive bibliography would have been useful.This article is a great faculty development resource and will serve as an excellent resource for all educators engaged in simulation based education.

Reviewer Expertise:

NA

No decision status is available
